# Prohibitin 2 ameliorates cisplatin-induced acute kidney injury by modulating mitochondrial homeostasis

**DOI:** 10.3389/fphys.2025.1658685

**Published:** 2025-12-08

**Authors:** Qi Zhang, Xiaoyu Shi, Lu Yao, Ping Zhou, Wei Ding

**Affiliations:** 1 Division of Nephrology, Shanghai Ninth People’s Hospital, School of Medicine, Shanghai Jiaotong University, Shanghai, China; 2 Department of Pediatric Nephrology and Rheumatology, Sichuan Provincial Maternity and Child Healthcare Hospital, Sichuan Clinical Research Center for pediatric nephrology, Chengdu, Sichuan, China

**Keywords:** acute kidney injury, cisplatin, mitochondrial dysfunction, prohibitin 2, renal protection

## Abstract

Acute kidney injury (AKI), associated with a major health burden globally, is frequently caused by nephrotoxic agents, specifically cisplatin. Prohibitin (PHB) 2, a highly conserved mitochondrial protein localized at the inner mitochondrial membrane, is key to maintaining mitochondrial respiration, cristae morphogenesis, and regulating cell death. Despite being extensively assessed in chronic kidney disease models, the role of PHB2 in AKI, particularly cisplatin-induced AKI, warrants further exploration. Here, we investigated the protective effects of PHB2 in cisplatin-induced AKI in in vitro and *in vivo* models. The results demonstrated that cisplatin upregulated PHB2 expression both *in vitro* and *in vivo*. Mechanistically, PHB2 deficiency exacerbated cisplatin-induced cell apoptosis and mitochondrial dysfunction, indicated by increased caspase-3 activity and reactive oxygen species (ROS) production, as well as mitochondrial membrane potential loss, *in vitro*. Our Western blot analysis results further validated PHB2’s involvement in autophagy processes within renal tubular cells. Nevertheless, PHB2 overexpression mitigated these detrimental effects, suggesting the protective role of PHB2 in cisplatin-induced AKI. *In vivo*, adeno-associated virus–mediated PHB2 overexpression reduced cisplatin-induced renal tubular injury and enhanced mitochondrial ultrastructure, supporting its potential therapeutic benefits. Taken together, our findings underscore the protective role of PHB2 in cisplatin-induced AKI, highlighting its potential as a therapeutic target for mitigating renal injury. Future studies elucidating the mechanisms underlying the protective effects of PHB2 and exploring its clinical implications in AKI management are warranted.

## 1 Introduction

Acute kidney injury (AKI) is a clinical syndrome characterized by the rapid decline in renal function and associated with a considerable health burden worldwide (Kurzhagen et al., 2020). AKI is often triggered by various insults, including nephrotoxic agent use, sepsis, and ischemia–reperfusion. In particular, cisplatin, a chemotherapeutic agent widely used for treating solid tumors, can induce nephrotoxicity in 20%–30% of patients undergoing chemotherapy. Cisplatin accumulation in renal tissues, particularly in the tubular epithelium, leads to cellular stress, followed by renal dysfunction (Volovat et al., 2023).

Cisplatin-induced AKI development involves multifaceted, complex mechanisms encompassing oxidative stress, inflammation, and cell death (Volovat et al., 2023). Notably, mitochondrial dysfunction is a pivotal player in cisplatin-induced renal tubular epithelial cell injury pathogenesis. The mitochondrion, the powerhouse of a cell, generates adenosine triphosphate (ATP) and maintains cellular homeostasis (Tang et al., 2021). However, cisplatin exposure disrupts mitochondrial integrity, promoting reactive oxygen species (ROS) production, impairing mitochondrial membrane potential, and inducing mitochondrial fission and fragmentation. These perturbations culminate in apoptosis, which thereby exacerbates renal injury (Tang et al., 2023).

Prohibitin (PHB) 2 is a highly conserved mitochondrial protein with diverse roles in mitochondrial biology and cellular homeostasis. PHB2 and its binding partner, PHB1, form a ring-like complex at the inner mitochondrial membrane. PHB2 participates in various cellular processes, including mitochondrial respiration, cristae morphogenesis, and cell death regulation. Notably, PHB2 selectively removes damaged mitochondria via autophagy (i.e., mitophagy), thus maintaining mitochondrial health and cellular homeostasis (Zhang et al., 2024). Although PHB2 has been detected in many chronic kidney disease models, its role in acute models, particularly cisplatin-induced AKI, requires further exploration (Liu et al., 2022; Wang et al., 2020; Xu et al., 2019). In this study, we explored the role of PHB2 in cisplatin-induced AKI, with a focus on its impact on mitochondrial homeostasis. We investigated the effects of PHB2 modulation on cisplatin-induced renal tubular epithelial cell apoptosis, mitochondrial dysfunction, and autophagy inhibition in both *in vitro* and *in vivo* models. Our findings provide insights into the protective mechanisms of PHB2 in AKI and highlight its potential as a therapeutic target for mitigating cisplatin-induced renal injury.

## 2 Materials and methods

### 2.1 Antibodies and reagents

Antibodies specific for Bcl-2-associated X protein (Bax; 2772), microtubule-associated protein light chain 3 (LC3; 12741T), cleaved caspase-3 (9661 for immunohistochemical staining), and β-tubulin (2128T) were procured from Cell Signaling Technology. A mouse monoclonal antibody targeting PHB2 (sc-133094) was sourced from Santa Cruz Biotechnology. Antibodies against B-cell lymphoma 2 (Bcl-2; ab182858), cleaved caspase-3 (ab32351 for Western blot), autophagy-related gene 7 (ATG7; ab133528), and P62 (ab56416) were procured from Abcam. Antibodies against β-actin (A20120A0702) and glyceraldehyde 3-phosphate dehydrogenase (GAPDH; A20120A0701) were obtained from BioTNT. All horseradish peroxidase (HRP)-conjugated secondary antibodies used for Western blot analysis were sourced from Jackson ImmunoResearch Laboratories. MitoSOX (M36008) was purchased from Thermo Fisher Scientific. A mitochondrial membrane potential assay kit containing JC-1 (C2003S) and ROS assay kit (S0033S) were obtained from Beyotime Biotechnology. A terminal deoxynucleotidyl transferase-mediated deoxyuridine triphosphate nick-end labeling (TUNEL) apoptosis detection kit (G1501) was sourced from Servicebio. Cisplatin (P4394) was acquired from Sigma-Aldrich.

### 2.2 Cell culture and transfection

HK-2 cells were maintained in Dulbecco’s Modified Eagle’s Medium (DMEM)/F-12 (SH30023; Hyclone), supplemented with 10% fetal bovine serum (04-001-1A; BioInd) at 37 °C in a humidified incubator under 5% CO_2_. PHB2-specific small interfering RNA (siRNA) and PHB2-overexpression (OE) plasmids were obtained from Genomeditech. After a 24-h incubation period in six-well plates, HK-2 cells were transfected with either 100 nM siRNA or 2 ng/mL plasmid using Lipo8000 (C0533; Beyotime Biotechnology) for an additional 48 h, according to the manufacturer’s protocol. Negative control groups were subjected to parallel transfection with scrambled siRNA or empty plasmid. The following siRNA sequences were used: 5′-CAUCACAGAAUCGUAUCUA-3′ and 5′-UAGAUACGAUUCUGUGAUG-3′. Subsequently, the HK-2 cells were exposed to 20 µM cisplatin for 24 h to induce cellular stress and assess the effects of PHB2 modulation on cisplatin-induced cellular responses.

### 2.3 Animals and adeno-associated virus infection

All animal experiments were performed in accordance with the National Institutes of Health’s *Guide for the Care and Use of Laboratory Animals* and approved by the Ethics Committee of Shanghai Ninth People’s Hospital. Eight-week-old male C57BL/6 mice were acquired from Shanghai Jihui Laboratory Animal Center and housed in standard facilities maintained at constant humidity and temperature, with *ad libitum* access to water and chow. The animals were randomly assigned to four groups (n = 6 per group). PHB2 overexpression was achieved through adeno-associated virus (AAV) serotype 9 (AAV9)-mediated gene transfer. AAV9 vectors encoding mouse PHB2 were provided by Taitool Bioscience. In brief, the C57BL/6 mice were anesthetized via inhalation of 2% i*soflurane*. They were then injected with 4.4 × 10^12^ viral genomes per milliliter of AAV2/9-CAG-PHB2-flag-WPRE-pA or AAV2/9-CAG-3×flag-WPRE-pA (control group) at five sites (10 μL per site) on the renal cortex by using a glass micropipette (1705RN; Hamilton). Renal injury was induced via a single intraperitoneal injection of cisplatin at a dose of 20 mg/kg body weight. Mice were euthanized at 28 days after AAV infection and 72 h after cisplatin injection via intraperitoneal administration of 150 mg/kg 3% pentobarbital sodium for further analysis.

### 2.4 Renal function, histopathology, and immunohistochemical staining

Harvested kidneys were fixed in 4% paraformaldehyde, embedded in paraffin, and sectioned into 3-μm-thick slices. These sections were hematoxylin and eosin (HE) and periodic acid-Schiff (PAS) staining. Tubular injury was scored according to the percentage of damaged tubules: 0, normal (no damaged tubule); 1, <25% damaged areas; 2, 25%–49% damaged areas; 3, 50%–74% damaged areas; and 4, ≥75% damaged areas. For immunohistochemical analyses, mouse kidneys were fixed in 4% paraformaldehyde for at least 24 h, embedded in paraffin, and sectioned. These sections were deparaffinized, hydrated in graded ethanol series, and stained using the peroxidase–antiperoxidase method. The sections were then incubated with the anti-PHB2 (1:100) and anti-cleaved caspase-3 (1:400) at 4 °C overnight and then with the corresponding secondary antibodies.

### 2.5 Transmission electron microscopy

Fresh kidney tissues were harvested and sectioned into 1-mm^3^ cubes. These cubes were prefixed in 2% glutaraldehyde, according to standard procedures. Next, the tissues were washed thoroughly, subjected to a dehydration process, and embedded in resin. The embedded tissues were sliced into ultrathin (70-nm-thick) sections by using standard methods. These sections were stained with lead citrate for enhanced contrast and visibility under the microscope. Finally, the stained, embedded sections were examined and detected on the transmission electron microscope FEI Talos L120C.

### 2.6 TUNEL assay for apoptosis

Apoptosis in cells was detected through the TUNEL assay, according to the manufacturer’s instructions. In brief, fixed and permeabilized HK-2 cells and paraffin-embedded sections were incubated with 56 µL of the TUNEL reaction mixture at 37 °C for 1 h in the dark. The samples were subsequently stained with 4′,6-diamidino-2-phenylindole for 8 min. TUNEL-positive cells within a 0.25-mm^2^ area were enumerated.

### 2.7 Mitochondrial ROS and mitochondrial membrane potential detection

Intracellular and mitochondrial ROS levels in viable HK-2 cells were measured using ROS Assay Kit and MitoSOX, according to the manufacturer’s instructions. In brief, cells were incubated with 10 µL of 2′, 7′-dichlorodihydrofluorescein diacetate (DCFH-DA) at 37 °C for 20 min or with 10 µL of 5 µM MitoSOX at 37 °C for 30 min in the dark. Mitochondrial transmembrane potential was evaluated using a JC-1 assay kit, as described previously (Xu et al., 2019). After treatment with cisplatin for 24 h, HK-2 cells were labeled with JC-1 (300 nM) at 37 °C for 20 min.

### 2.8 Real-time polymerase chain reaction

Total RNA was isolated and reverse-transcribed by using a PrimeScript RT reagent kit (Takara), according to the manufacturer’s protocol. Real-time polymerase chain reaction (PCR) was performed on an ABI Prism 7,500 sequence detection system, and the relative expression levels of the target genes were normalized against the expression of GAPDH or 18s ribosomal RNA (rRNA) as an internal control. The specific primer sequences employed for the PCR reactions were as follows: mitochondrial ATP synthase 6 (mt-Atp6), 5′-AGGACGAACATGAACCCTAAT-3′ and 5′-CAGCTCATAGTGGAATGGCTA-3′ for mouse; mitochondrial cytochrome b (mt-Cytb), 5′-CCCAGACAACTACATACCAGC-3′ and 5′-GATTAAGGCTAGGACACCTCC-3′ for mouse; 18s rRNA, 5′-GTCTCAAAGATTAAGCCATGC-3′ and 5′-GACCAAAGGAACCATAACTGA-3′ for mouse; interleukin-6 (IL-6), 5′-AACAACCTGAACCTTCCAAAG-3′ and 5′-CAAACTCCAAAAGACCAGTGA-3′ for cell; IL-6, 5′-TAGTCCTTCCTACCCCAATTTCC-3′; 5′-TTGGTCCTTAGCCACTCCTTC-3′for mouse; tumor necrosis factor-α (TNF-α), 5′-CCAGGCAGTCAGATCATCTTC-3′ and 5′-GCTTGAGGGTTTGCTACAACA-3′ for cell; TNF-α, 5′-CCCTCACACTCAGATCATCTTCT-3′; 5′-GCTACGACGTGGGCTACAG-3′ for mouse; GAPDH, 5′-GACACTGAGCAAGAGAGGCCCTA-3′ and 5′-TGGATGAAATTGTGAGGA-3′ for mouse and GAPDH 5′-CCTCTGACTTCAACAGCGACA-3′ and 5′-ATGAGCTTGACAAAGTGGTCGT-3′ for cell.

### 2.9 Western blot analysis

Kidney cells were collected and lysed in radioimmunoprecipitation assay lysis buffer (Beyotime Biotechnology), according to the manufacturer’s protocol. All protein samples were separated through 10%–12% sodium dodecyl sulfate polyacrylamide gel electrophoresis and then transferred onto polyvinylidene fluoride membranes. After they were blocked with 5% nonfat milk for 2 h, the membranes were incubated with primary antibodies at 4 °C overnight. The membranes were subsequently incubated with secondary antibodies at room temperature for 1 h. Immunoreactive band intensities were visualized based on enhanced chemiluminescence, and densitometric analyses for quantification were performed using Image J.

### 2.10 Statistical analysis

All data, presented as means ± standard deviations (SDs), were analyzed using one-way ANOVA for comparisons. A *P*-value of <0.05 was deemed to indicate statistical significance.

## 3 Results

### 3.1 Kidney PHB2 upregulation is associated with AKI

**FIGURE 1:**
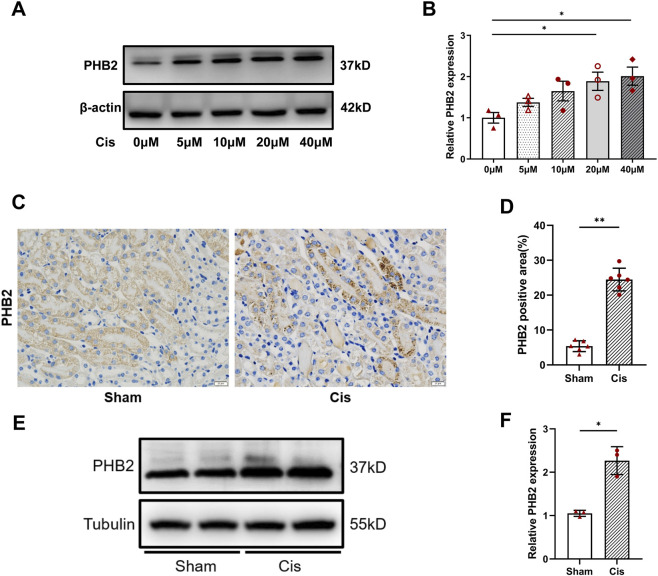
PHB2 expression was upregulated in mouse kidneys and renal tubular epithelial cells treated with cisplatin. **(A)** Western blots of PHB2 in renal tubular epithelial cells stimulated by different cisplatin concentrations (0–40 μM). **(B)** Densitometric analyses of PHB2 normalized to β-actin. **(C,D)** Immunohistochemical staining **(C)** and quantitative results **(D)** of kidney sections from mice treated with cisplatin. Scale bar, 20 μm. **(E,F)** Western blot analyses of protein expression **(E)** and densitometric analyses **(F)** of PHB2. Data are presented as means ± SDs (n = 3 or 6). *P < 0.05. **P < 0.01.

Our Western blot analysis revealed that after 24-h *in vitro* stimulation with cisplatin at varying concentrations, HK2 cells exhibited a gradual increase in PHB2 expression with an increase in cisplatin concentrations (Figures 1A,B). This finding was further corroborated by our *in vivo* observations: immunohistochemical staining of mouse renal tissues revealed PHB2 deposition in renal tubules after cisplatin treatment (Figures 1C,D). Similarly, intraperitoneal injection of cisplatin significantly increased PHB2 expression in mice (Figures 1E,F).

### 3.2 Cisplatin induces renal tubular cell apoptosis and inhibits autophagy

**FIGURE 2:**
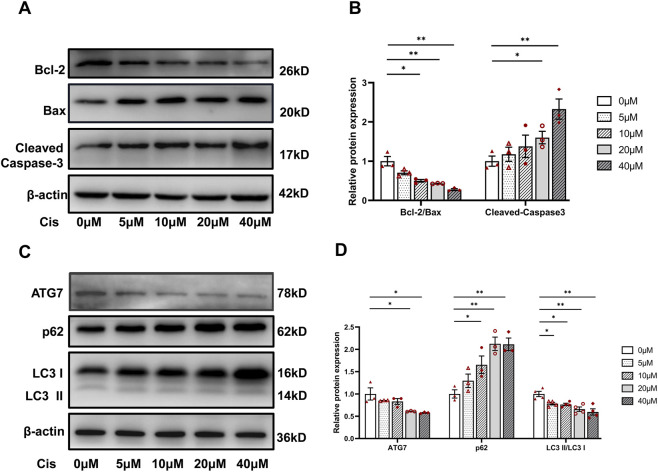
Cisplatin induced apoptosis and autophagy in renal tubular epithelial cells. **(A,B)** Western blots **(A)** and densitometric analyses **(B)** of Bcl-2, Bax, and cleaved Caspase-3 in renal tubular epithelial cells stimulated by cisplatin at different concentrations. **(C,D)** Western blots **(C)** and densitometric analyses **(D)** of ATG7, p62, and LC3 in renal tubular epithelial cells stimulated by cisplatin at different concentrations. Data are presented as means ± SDs (n = 3 or 4). *P < 0.05. **P < 0.01.

Western blot analysis of HK2 cells stimulated with cisplatin at different concentrations for 24 h revealed a significant increase in the expression of apoptosis-related proteins such as cleaved caspase-3 and Bax but a decrease in Bcl-2 expression along with a corresponding decline in the Bcl/Bax ratio (Figures 2A,B). These changes were consistent with the pathological manifestations of renal tubular necrosis observed in our cisplatin-treated mice (Figure 1C). Moreover, autophagy-related proteins exhibited dose-dependent changes in expression: as the cisplatin concentration increased, the LC3 II/LC3 I ratio and ATG7 level decreased and P62 levels increased (Figures 2C,D).

### 3.3 PHB2 deficiency exacerbates cisplatin-induced cell apoptosis

**FIGURE 3:**
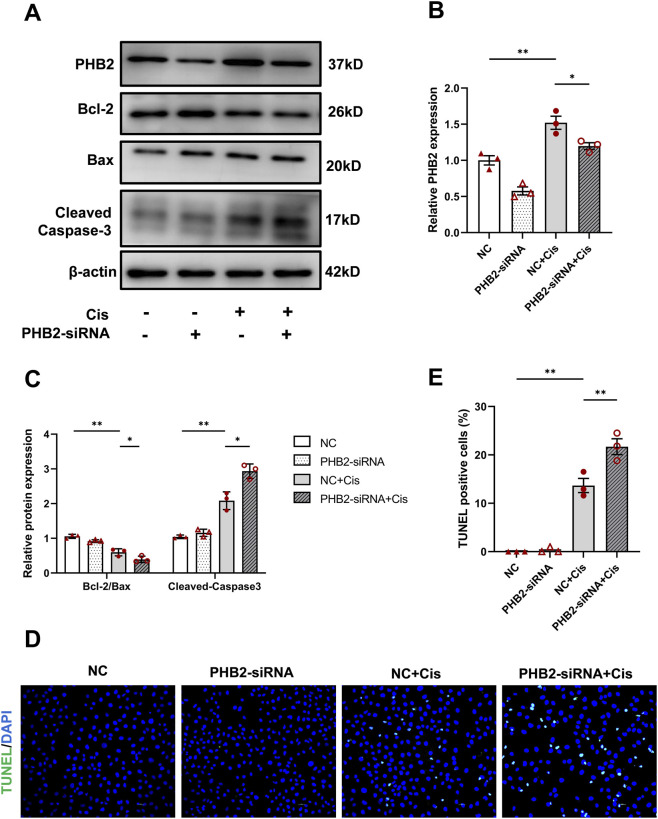
PHB2 silencing exacerbated apoptosis in cisplatin-treated HK-2 cells. **(A–C)** Western blots **(A)** and quantitative results **(B,C)** of PHB2, Bcl-2, Bax, and cleaved caspase-3 in HK-2 cells transfected with PHB2-siRNA and treated with 20 μM cisplatin for 24 h. **(D)** TUNEL staining for apoptosis evaluation. **(E)** Quantification of TUNEL-positive cells. Scale bar, 10 μm. Data are presented as means ± SDs (n = 3). *P < 0.05. **P < 0.01.

Transfection of HK2 cells with *PHB2*-siRNA significantly reduced PHB2 levels (Figures 3A,B). After subsequent stimulation with 20 μmol/L cisplatin for 24 h, PHB2 siRNA transfection significantly exacerbated cisplatin-induced cell apoptosis, as indicated by increased expression of cleaved caspase-3 and Bax and decreased Bcl-2 expression, along with a decline in the Bcl/Bax ratio to the lowest level among all groups (Figures 3A,C). TUNEL assay also revealed increased tubular cell apoptosis in the PHB2-deficient group after cisplatin stimulation, consistent with our Western blot analysis results (Figures 3D,E).

### 3.4 PHB2 deficiency exacerbates cisplatin-induced mitochondrial damage in renal tubular cells

**FIGURE 4:**
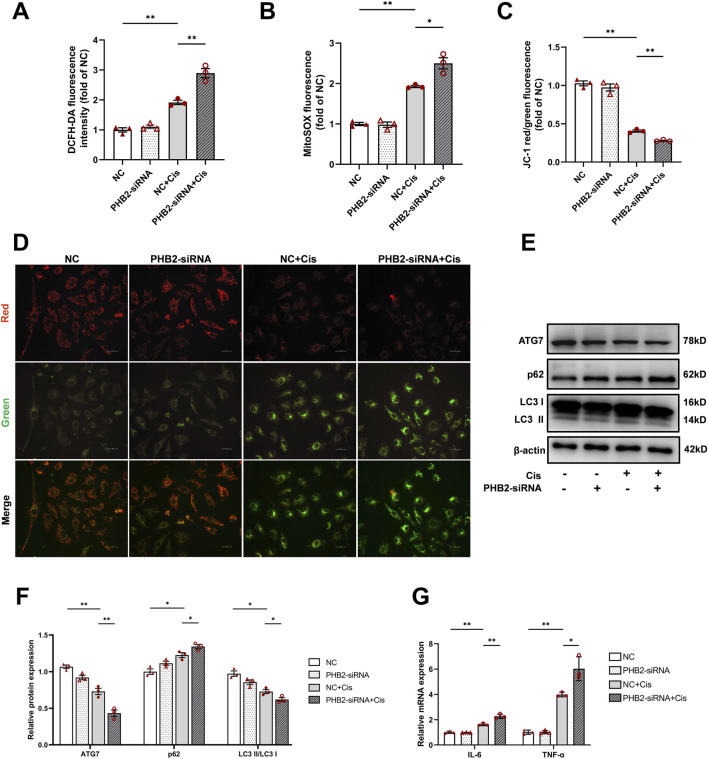
PHB2 silencing aggravated cisplatin-induced mitochondrial dysfunction and inflammation in HK-2 cells. **(A)** Quantification of fluorescence in DCFH-DA-stained HK-2 cells. **(B)** Quantification of fluorescence of MitoSOX-stained HK-2 cells. **(C,D)** Representative images of HK-2 cells subjected to JC-1 staining **(D)** and quantification of red and green fluorescence **(C)**. Scale bar, 25 μm. **(E,F)** Western blots **(E)** and quantification **(F)** of ATG7, p62, and LC3 in HK-2 cells transfected with *PHB2*-siRNA and treated with 20 μM cisplatin for 24 h. **(G)** Changes in the mRNA levels of IL-6 and TNF-α following cisplatin stimulation and siRNA transfection. Data are presented as means ± SDs (n = 3). *P < 0.05. **P < 0.01.

Our DCFH-DA and MitoSOX staining results demonstrated that cisplatin stimulation increased both intracellular and mitochondrial ROS production, which was further elevated by *PHB2*-siRNA transfection; thus, PHB2 deficiency promotes ROS production (Figures 4A,B). JC-1 staining revealed a significant shift from red to green fluorescence in the group transfected with *PHB2*-siRNA and stimulated with cisplatin, indicating a severe loss of mitochondrial membrane potential (Figures 4C,D). Western blot analysis further revealed that *PHB2*-siRNA transfection led to a further reduction in the LC3 II/LC3 I ratio and ATG7 levels and an increase in P62 levels compared with the cisplatin-only group. Therefore, PHB2 deficiency exacerbated autophagy inhibition (Figures 4E,F). Moreover, the lack of PHB2 resulted in a further elevation of the inflammatory cytokines IL-6 and TNF-α upon cisplatin stimulation, suggesting a potential link between PHB2 and the regulation of inflammation (Figure 4G).

### 3.5 PHB2 overexpression ameliorates cisplatin-induced renal tubular cell apoptosis

**FIGURE 5:**
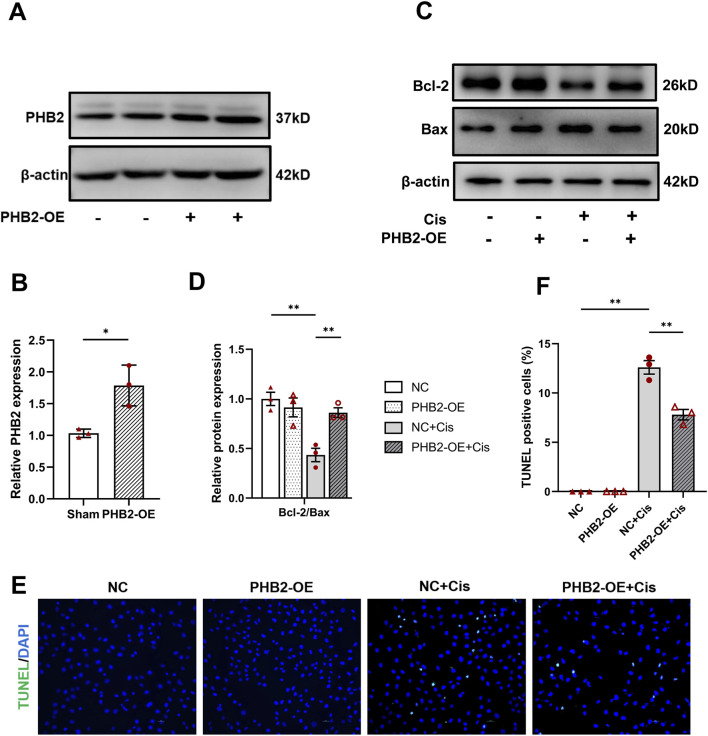
PHB2 overexpression ameliorated cisplatin-induced AKI in HK2 cells. **(A–D)** Western blots **(A,C)** and quantification **(B,D)** of PHB2, Bcl-2 and Bax in HK-2 cells transfected with PHB2-OE plasmid and treated with 20 μM cisplatin for 24 h. **(E)** TUNEL assay for apoptosis. **(F)** Quantification of TUNEL-positive cells. Scale bar, 10 μm. Data are presented as means ± SDs (n = 3). *P < 0.05. **P < 0.01.

Transfection of HK2 cells with a *PHB2*-OE plasmid (Figures 5A,B) significantly reversed the increases in Bax expression and decreases in Bcl-2 expression induced by cisplatin (Figures 5C,D). Moreover, the TUNEL assay revealed that *PHB2*-OE plasmid transfection effectively reduced HK2 cell apoptosis caused by cisplatin, consistent with our Western blot analysis results (Figures 5E,F). Thus, PHB2 overexpression had a protective effect on renal tubules.

### 3.6 PHB2 overexpression ameliorates cisplatin-induced mitochondrial homeostasis imbalance

**FIGURE 6:**
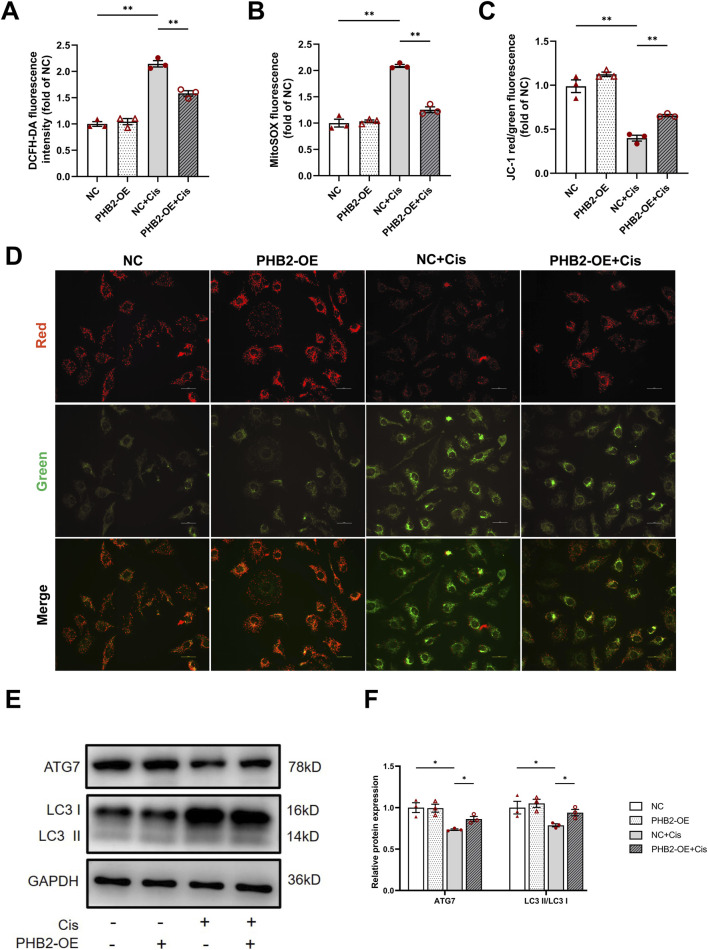
PHB2 overexpression attenuated cisplatin-induced mitochondrial dysfunction *in vitro*. **(A)** Quantification of fluorescence in DCFH-DA-stained HK-2 cells. **(B)** Quantification of fluorescence of MitoSOX-stained HK-2 cells. **(C,D)** Representative images of HK-2 cells subjected to JC-1 staining **(D)** and quantification of red and green fluorescence **(C)**. Scale bar, 25 μm. **(E,F)** Western blots **(E)** and quantification **(F)** of ATG7 and LC3 in HK-2 cells transfected with *PHB2*-OE plasmid and treated with 20 μM cisplatin for 24 h. Data are presented as means ± SDs (n = 3). *P < 0.05. **P < 0.01.

DCFH-DA and MitoSOX staining revealed that PHB2-OE plasmid transfection effectively reduced cisplatin-induced cellular and mitochondrial ROS accumulation (Figures 6A,B). Moreover, JC-1 staining directly demonstrated alterations in mitochondrial membrane potential, with increased red fluorescence intensity and decreased green fluorescence intensity in the group with PHB2 overexpression followed by cisplatin stimulation. Thus, PHB2 overexpression reversed the loss of mitochondrial membrane potential caused by cisplatin (Figures 6C,D). Western blot analysis further showed that PHB2 overexpression improved cisplatin-induced autophagy inhibition, as evidenced by increased LC3 II/LC3 I ratio and ATG7 expression (Figures 6E,F).

### 3.7 AAV-mediated PHB2 overexpression ameliorates cisplatin-induced AKI in mice

**FIGURE 7:**
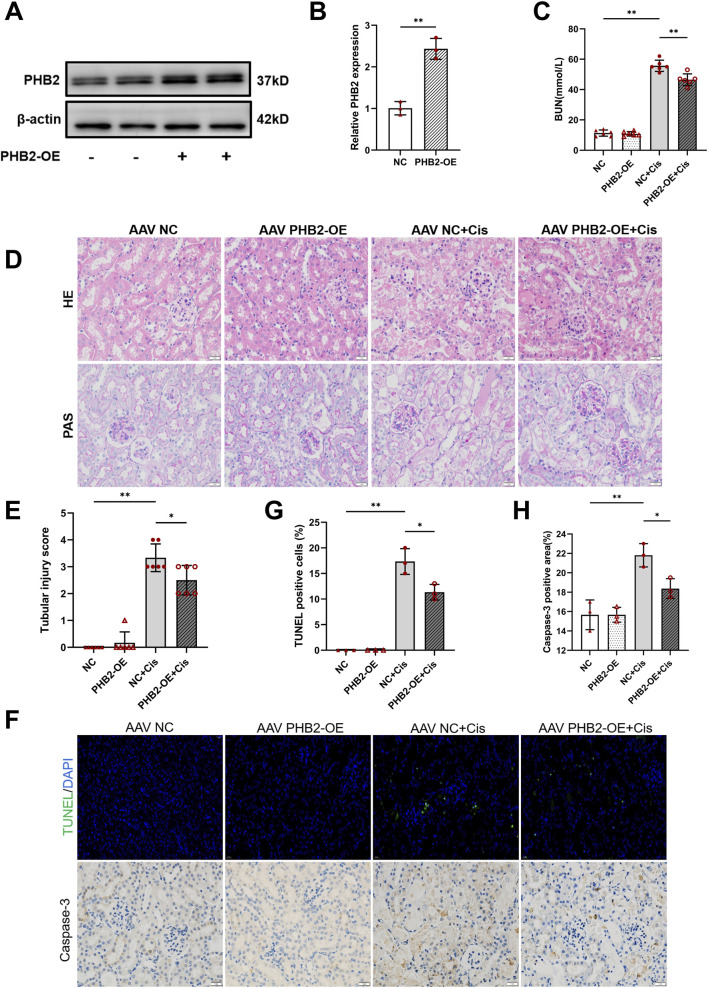
PHB2 overexpression in kidneys protected against cisplatin-induced AKI. **(A,B)** Western blots **(A)** and quantification **(B)** of PHB2 after AAV injection. **(C)** Serum blood urea nitrogen levels. **(D)** Kidney sections subjected to HE and PAS staining. Scale bar, 1 mm. **(E)** Statistical analysis of tubular injury scores. **(F)** Apoptosis was evaluated by TUNEL assay (scale bar, 25 μm) and immunohistochemical staining of cleaved caspase-3 in kidneys (scale bar, 1 mm). **(G,H)** Quantification of TUNEL-positive cells **(G)** and cleaved caspase-3-positive areas **(H)**. Data are presented as means ± SDs (n = 3 or 6). *P < 0.05. **P < 0.01.

C57BL/6 mice were locally injected with AAV into one kidney and sacrificed 1 month after injection. Before euthanasia, the mice were administered 20 mg/kg cisplatin intraperitoneally. Western blot revealed a significant increase in *PHB2* protein expression after local AAV injection (Figures 7A,B). Paraffin-embedded renal tissue sections were subjected to HE and PAS staining. The results demonstrated that AAV injection significantly mitigated cisplatin-induced loss of brush border, cast formation, lumen dilation of renal tubules, and tubular epithelial cell degeneration (Figure 7D), accompanied by a corresponding reduction in the tubular injury score (Figure 7E). AAV injection also attenuated the cisplatin-induced elevation in urea nitrogen levels (Figure 7C). Furthermore, TUNEL assay and immunohistochemical staining for cleaved caspase-3 indicated that PHB2 overexpression effectively reduced cellular apoptosis (Figures 7F–H). Taken together, these findings suggested that PHB2 overexpression effectively mitigates cellular apoptosis, preserves renal function, and alleviates cisplatin-induced AKI.

### 3.8 AAV-mediated PHB2 overexpression ameliorates cisplatin-induced mitochondrial homeostasis imbalance in mice

**FIGURE 8:**
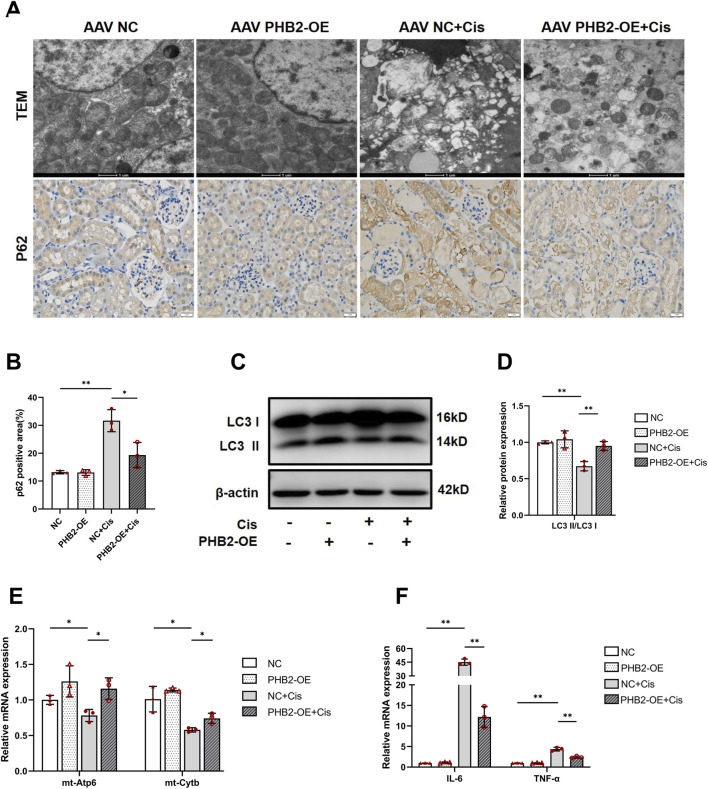
PHB2 overexpression attenuated cisplatin-induced mitochondrial dysfunction and inflammation *in vivo*. **(A)** Representative transmission electron microscopy images of mitochondria in renal tubular epithelial cells (scale bar, 1 μm) and immunohistochemical staining of p62 in kidneys (scale bar, 1 mm). **(B)** Quantification of p62 positive area. **(C,D)** Western blots **(C)** and quantification **(D)** of LC3 after AAV injection. **(E)** Relative levels of mt-Atp6 and mt-Cytb mRNAs in the kidneys. **(F)** mRNA levels of IL-6 and TNF-α. Data are presented as means ± SDs (n = 3). *P < 0.05. **P < 0.01.

Transmission electron microscopy revealed mitochondrial swelling and cristae loss in renal tubular cells of mice treated with cisplatin. Notably, AAV-mediated PHB2 overexpression amended these mitochondrial abnormalities (Figure 8A). Furthermore, Western blot analysis and immunohistochemical staining revealed that PHB2 overexpression reversed cisplatin-induced autophagy inhibition, marked by an increased LC3 II/LC3 I ratio and downregulation of p62 (Figures 8A–D). Concurrently, cisplatin exposure significantly reduced the RNA levels of genes related to mitochondrial energy metabolism (Figure 8E). PHB2 overexpression ameliorated mitochondrial damage caused by cisplatin. Consistent with the *in vitro* findings, PHB2 overexpression in mouse kidneys also alleviated the cisplatin-induced inflammatory response (Figure 8F).

## 4 Discussion

Cisplatin is widely used in chemotherapy regimens for the treatment of various solid tumors. Because it is mainly excreted through glomerular filtration and tubular excretion, cisplatin can accumulate in the kidneys. In 20%–30% of all patients, chemotherapy based on cisplatin causes nephrotoxicity (Arany and Safirstein, 2003). Cisplatin is metabolically activated to its toxic forms after uptake by renal tubular cells. Cisplatin accumulation in renal proximal tubular cells induces DNA damage, mitochondrial damage, oxidative stress, endoplasmic reticulum stress, and autophagy, leading to renal cell inflammation and cellular senescence. This can eventually result in renal tubular cell injury and AKI (Tang et al., 2023). Effective therapeutic targets for cisplatin-induced kidney injury, particularly in the mitochondria involved in energy metabolism, are needed urgently.

PHBs belong to the stomatin/prohibitin/flotillin/HflKC family, and the human genome encodes two PHBs: PHB1 and PHB2. These PHBs not only function independently but also act as heterodimeric complexes. PHB2, expressed in the nucleus, mitochondria, and cytoplasm, has been noted to play crucial roles in models of various kidney injuries, such as diabetic nephropathy (Liu et al., 2022), angiotensin II-induced kidney injury (Xu et al., 2019), and ischemia–reperfusion injury (Wang et al., 2020). In recent years, PHB2 has attracted considerable attention because of its multiple functions in mitochondria; however, its role in cisplatin-induced AKI has not been specifically reported thus far. Therefore, in the current study, we explored the role of PHB2 in cisplatin-induced AKI and the associated mechanisms.

Cisplatin-induced nephrotoxicity can involve various cell death modes, including apoptosis, necrosis, pyroptosis, and ferroptosis. In the mitochondria-mediated intrinsic apoptosis pathway, Bak and Bax are activated first. Next, porous defects occur in the mitochondrial outer membrane, and cytochrome C is released into the cytoplasm; this leads to the activation of caspase-9 and its downstream proteins (Tang et al., 2023). In the current study, we confirmed that 10–40 μmol/L cisplatin induced apoptosis alongside concentration-dependent increases in Bax and caspase-3 expression (Tang et al., 2023). In HK2 cells, PHB2 overexpression significantly reduced apoptosis, whereas *PHB2*-siRNA transfection increased caspase-3 expression. Similarly, in our mouse model, PHB2 overexpression induced by exogenous AAV ameliorated cisplatin-induced renal impairment and tubular injury; TUNEL assay and caspase-3 levels confirmed the protective effects of PHB2. Studies have reported that PHB2 protects cells against apoptosis not only by directly binding to antiapoptotic proteins such as HCLS1-associated protein X-1 (Kasashima et al., 2006; Shi et al., 2020) but also by modulating the mitochondrial structure and regulating cellular functions such as autophagy and oxidative phosphorylation.

Numerous GTPases mediate mitochondrial fission and fusion. The mitochondrial dynamin-like GTPase optic atrophy 1 protein (OPA1) is crucial in mitochondrial fusion and cristae structural remodeling. The balance between its two forms, the uncut long OPA1 (L-OPA1) and the cleaved short OPA1 (S-OPA1), determines cell fate (Del Dotto et al., 2017). The formation of a PHB1–PHB2 complex can stabilize OPA1, and *PHB2* knockdown can result in selective deletion of L-OPA1, accompanied by severe mitochondrial dysfunction and abnormal cristae morphogenesis (Li et al., 2019). In addition, PHB2 can influence the maturation and distribution of cardiolipin, affecting mitochondrial shape and ultrastructure (Richter-Dennerlein et al., 2014). Here, we observed that cisplatin stimulation led to mitochondrial swelling and cristae loss in renal mitochondria of mice; nevertheless, PHB2-overexpressing mice demonstrated reversal of cisplatin-induced mitochondrial damage.

PHB2 is pivotal in autophagy, particularly mitophagy (Galluzzi et al., 2017; Wei et al., 2017). After mitochondrial damage, PHB2, located at the inner mitochondrial membrane, becomes exposed and functions as a receptor for autophagy through its interaction with LC3, a key protein associated with autophagosome formation. This interaction is facilitated by the rupture of the outer mitochondrial membrane via a proteasome-dependent process, allowing PHB2 to bind to LC3 and participate in mitophagy (Wei et al., 2017). Furthermore, PHB2 is involved in ubiquitin-mediated autophagosome recruitment, where it acts as both a receptor and a recruitment protein (Lazarou et al., 2015; Sun et al., 2022). Studies have reported that PHB2 interacts with proteins, such as phosphatase and tensin homolog-induced kinase 1, Parkin, and Golgi phosphoprotein 3, influencing mitophagy and potentially linking Golgi apparatus activity to the aforementioned process (Bertolin et al., 2021; De Falco et al., 2020; Wang et al., 2021). Moreover, PHB2-mediated autophagy can occur independent of LC3 and involve autophagy receptors such as nuclear dot protein 52 and optineurin (Lazarou et al., 2015). Thus, PHB2 is a critical mediator of mitochondrial autophagy, influencing cellular homeostasis. In our *in vivo* and *in vitro* models of cisplatin-induced AKI, we confirmed the importance of PHB2 in maintaining mitochondrial homeostasis. *PHB2*-siRNA increased abnormal mitochondrial membrane potential, along with elevated levels of intracellular and mitochondrial ROS and altered expression of autophagy-associated proteins, such as LC3, ATG7, and P62. In contrast, in HK2 cells, PHB2 overexpression effectively ameliorated cisplatin-induced mitochondrial damage, reduced ROS accumulation, maintained undetermined membrane potential, and increased autophagy inhibition. In addition, we confirmed the role of PHB2 in maintaining mitochondrial stability at the morphological, protein, and mRNA levels in AAV-injected PHB2-overexpressing mice.

In conclusion, our findings revealed that cisplatin induces renal tubular epithelial dysfunction, leading to AKI, and that PHB2 can mitigate renal tubular epithelial cell apoptosis by modulating mitochondrial homeostasis. Thus, because of its protective effect against cisplatin-induced AKI, PHB2 is a potential therapeutic target for AKI management.

## Data Availability

The raw data supporting the conclusions of this article will be made available by the authors, without undue reservation.
