# Supplementary material for: Prohibitin 2 ameliorates cisplatin-induced acute kidney injury by modulating mitochondrial homeostasis
Source: Front Physiol. 2025 Dec 8;16:1658685. doi: 10.3389/fphys.2025.1658685 (PMC12719072; doi:10.3389/fphys.2025.1658685)
Supplement: Supplementary file 1 [file Presentation1.pptx]

## Slide 1
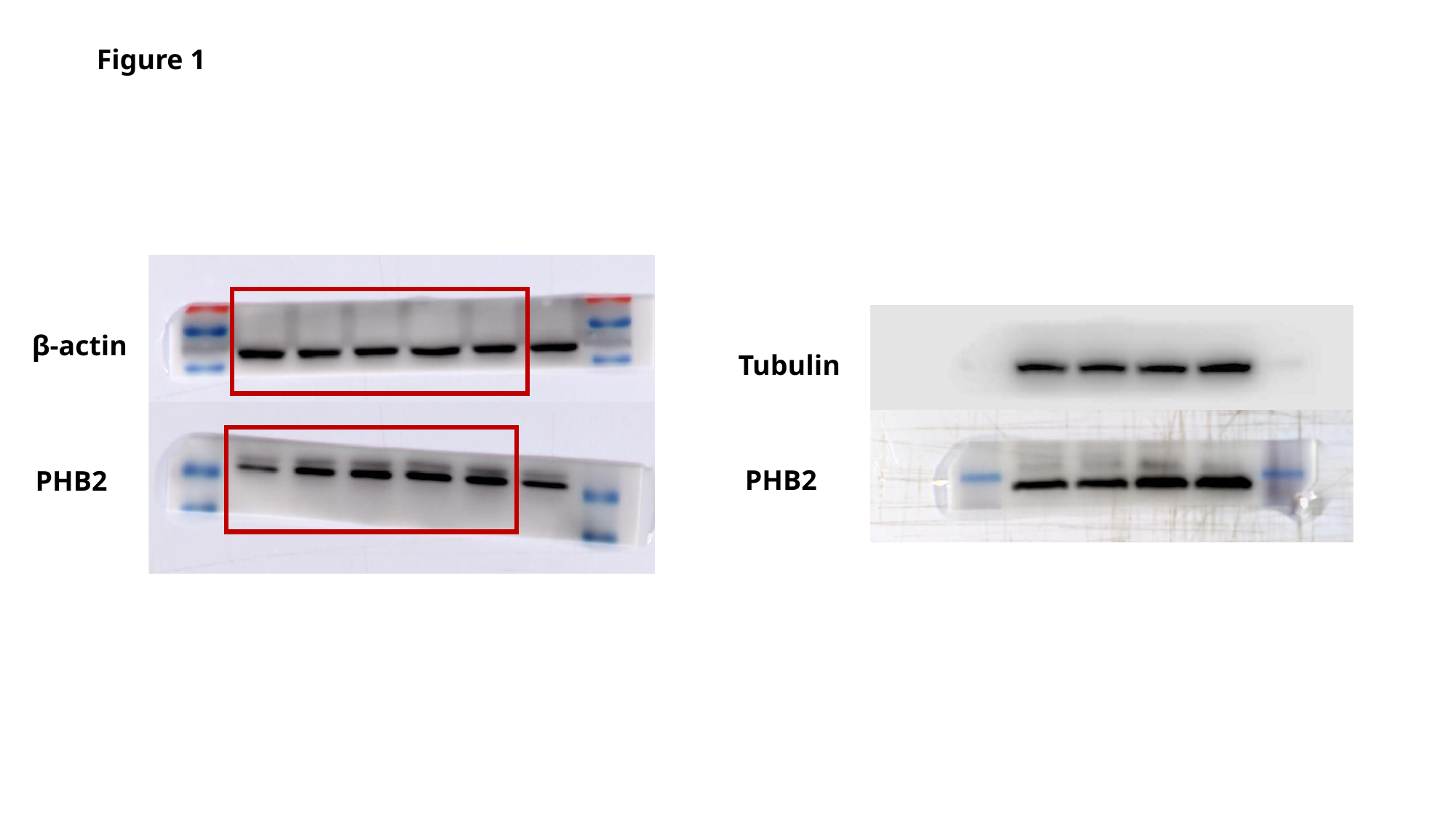

Figure 1
β-actin
Tubulin
PHB2
PHB2

## Slide 2
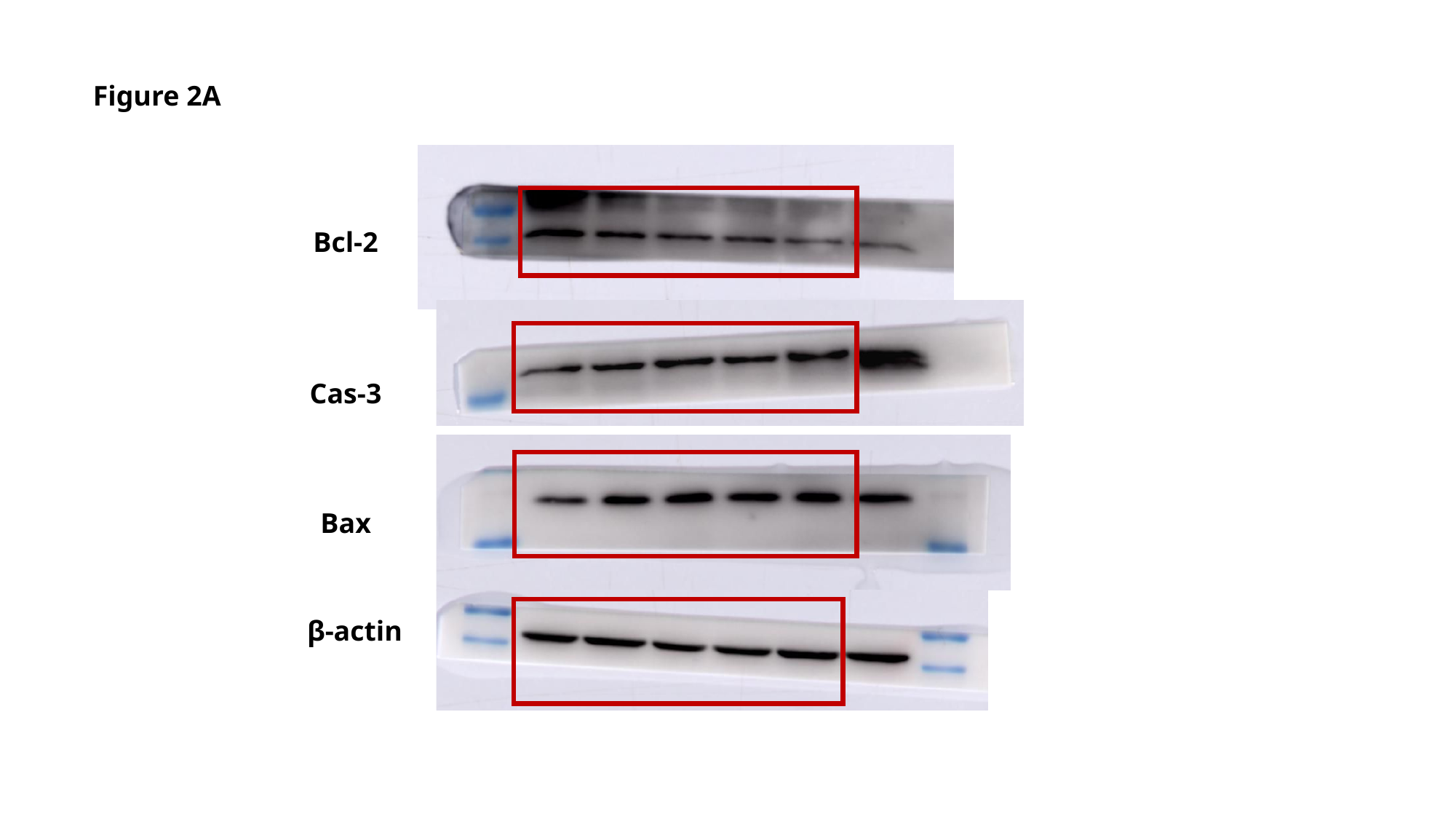

Figure 2A
Bcl-2
Cas-3
Bax
β-actin

## Slide 3
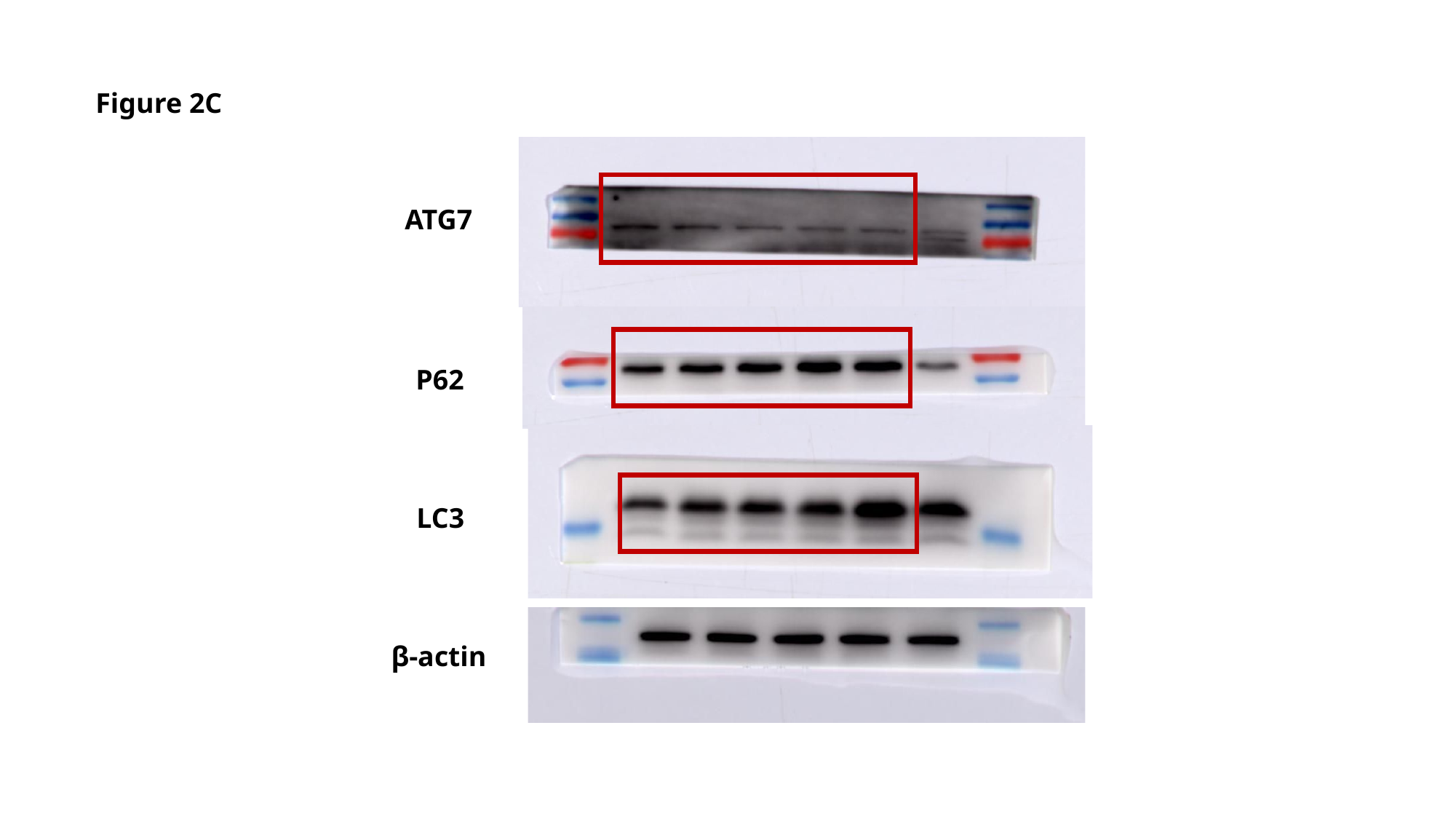

Figure 2C
ATG7
P62
LC3
β-actin

## Slide 4
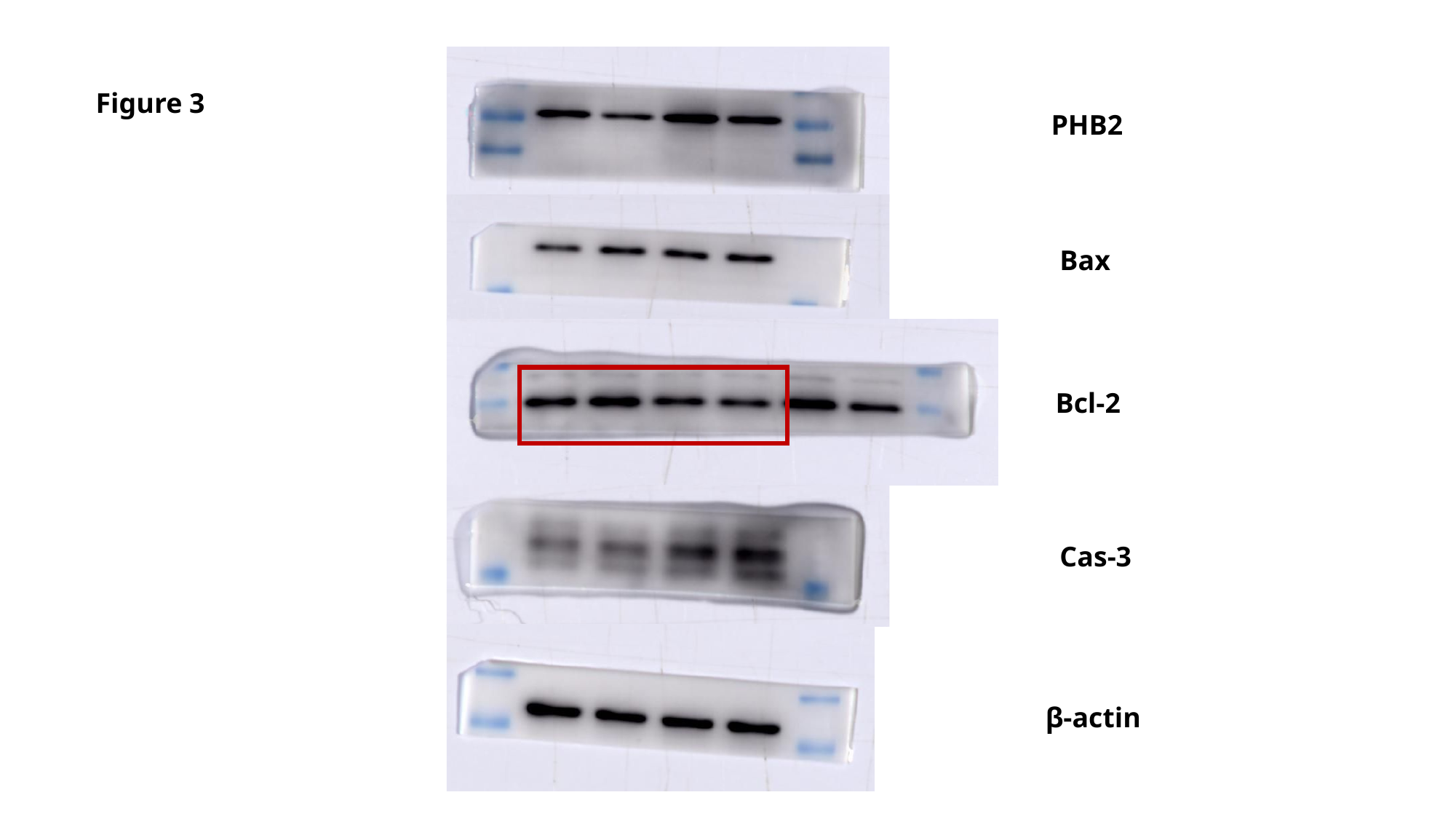

Figure 3
PHB2
Bax
Bcl-2
 Cas-3
β-actin

## Slide 5
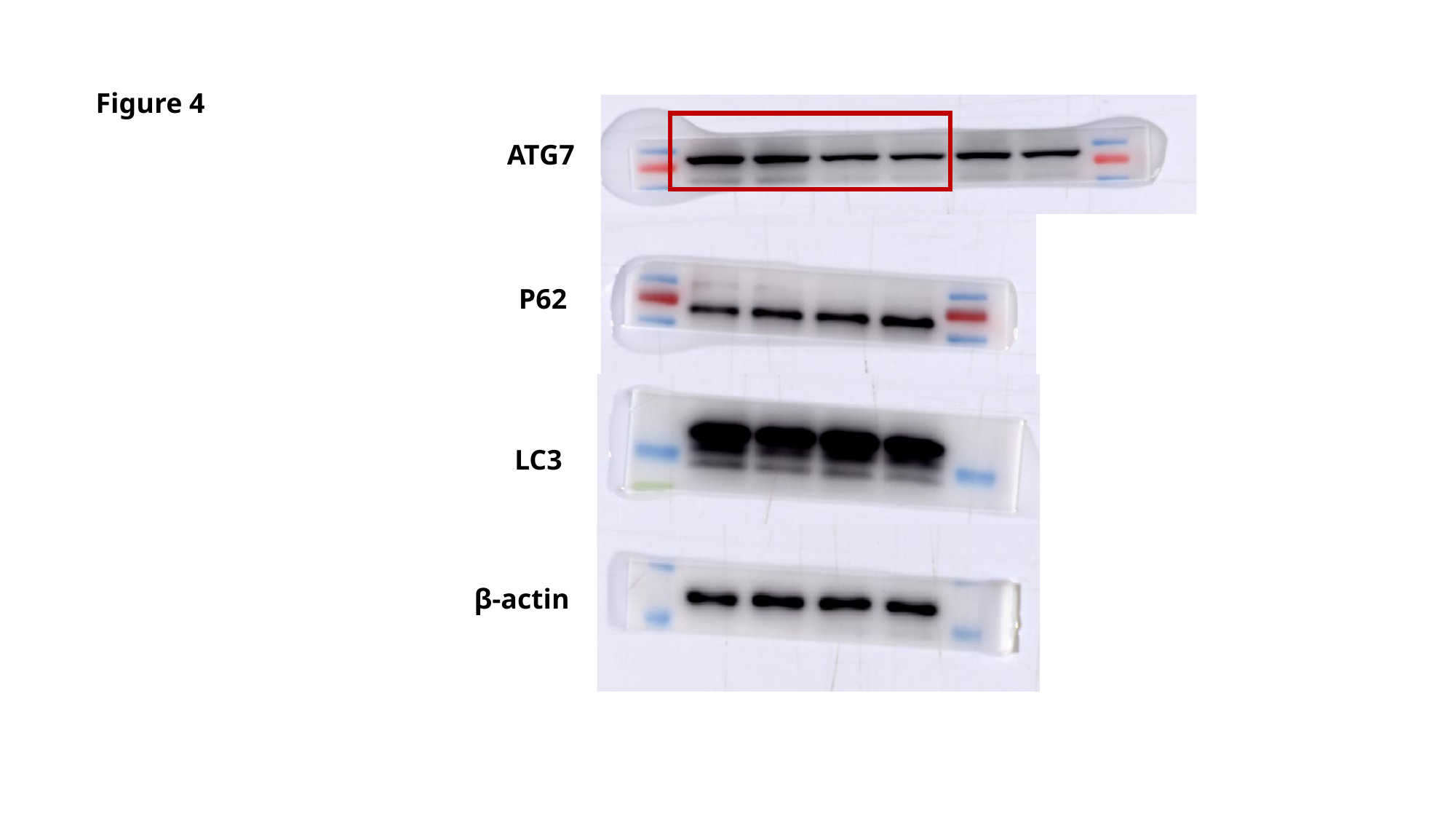

Figure 4
 ATG7
 P62
LC3
β-actin

## Slide 6
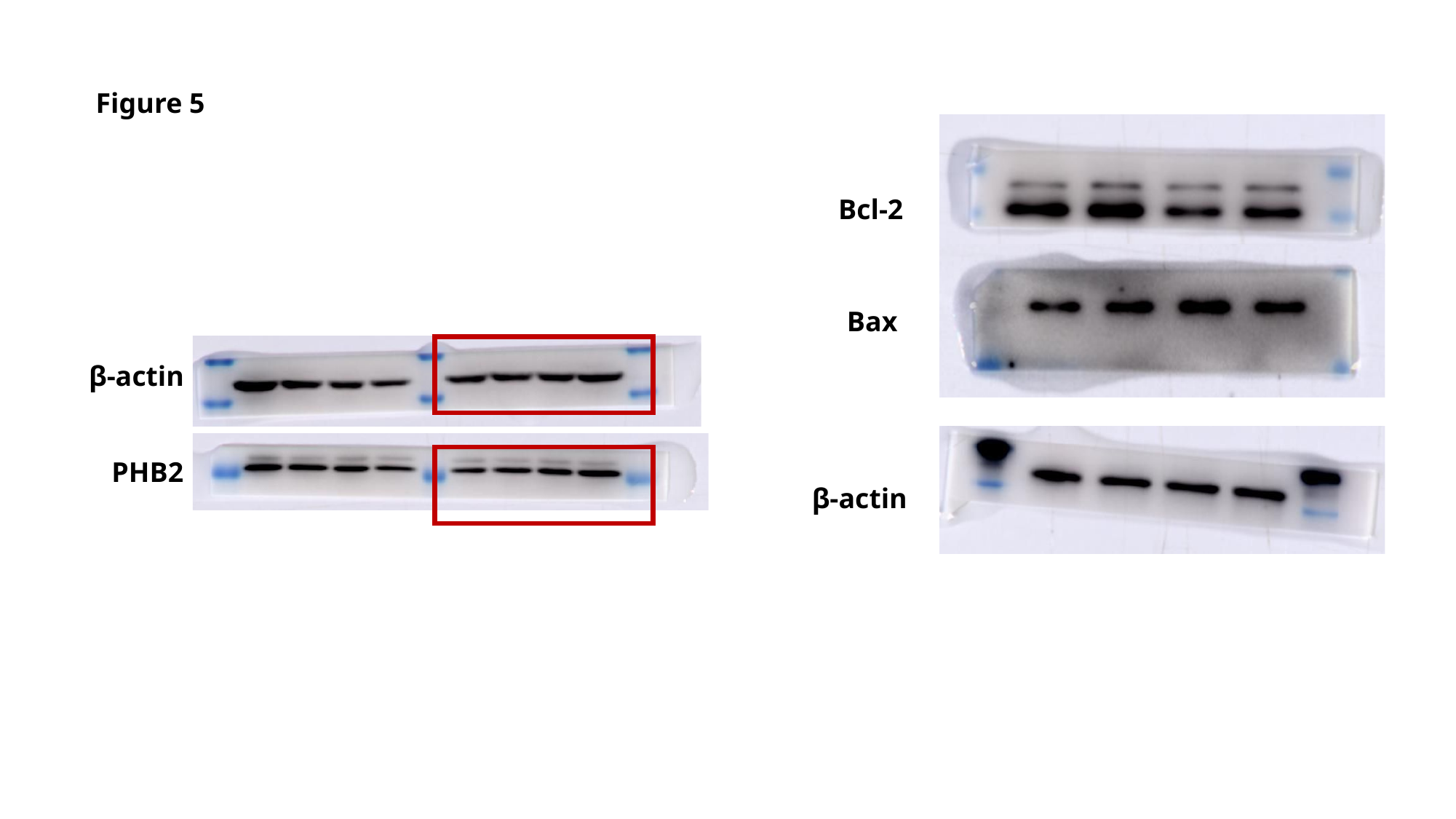

Figure 5
Bcl-2
Bax
β-actin
PHB2
β-actin

## Slide 7
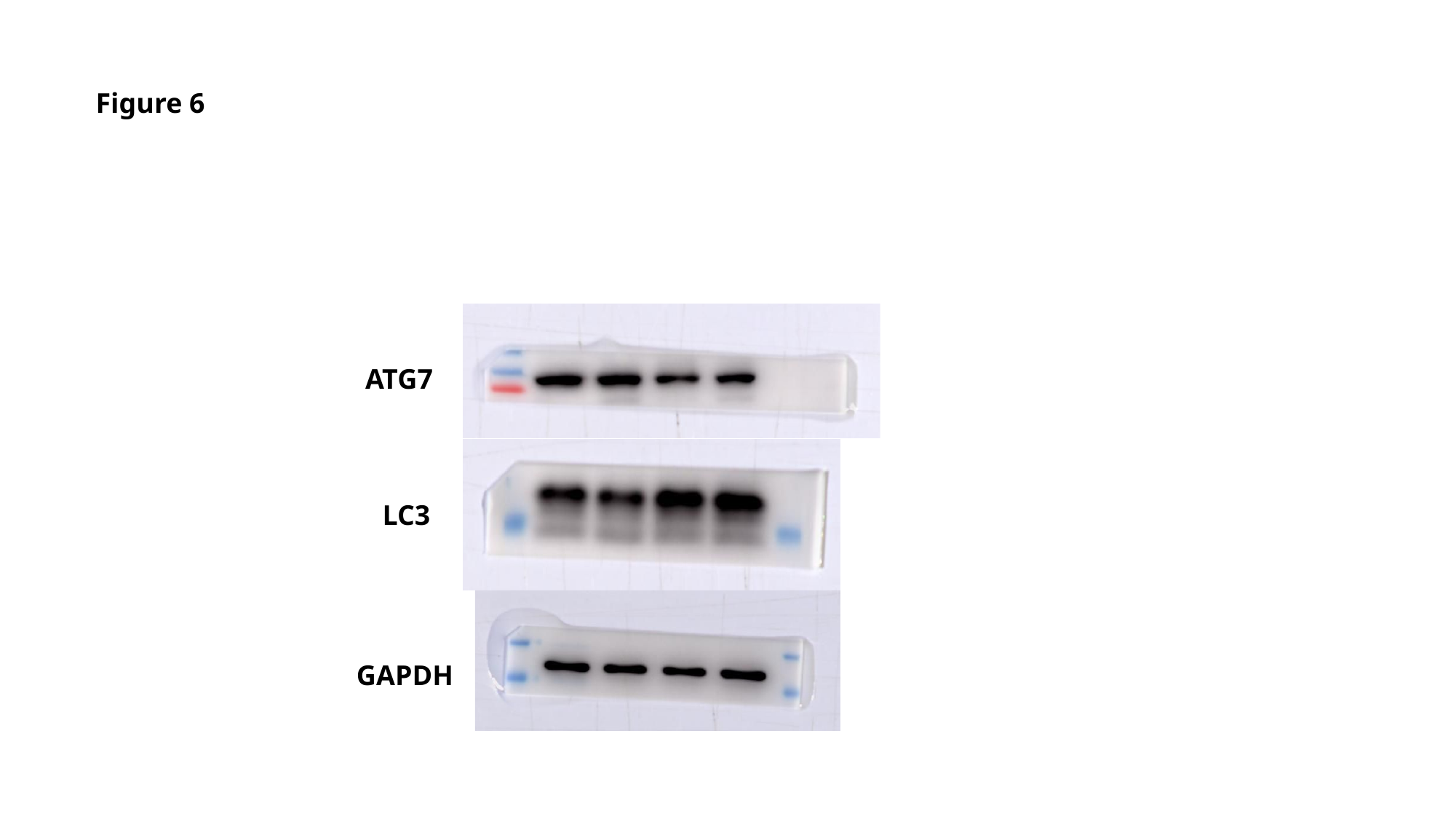

Figure 6
ATG7
LC3
GAPDH

## Slide 8
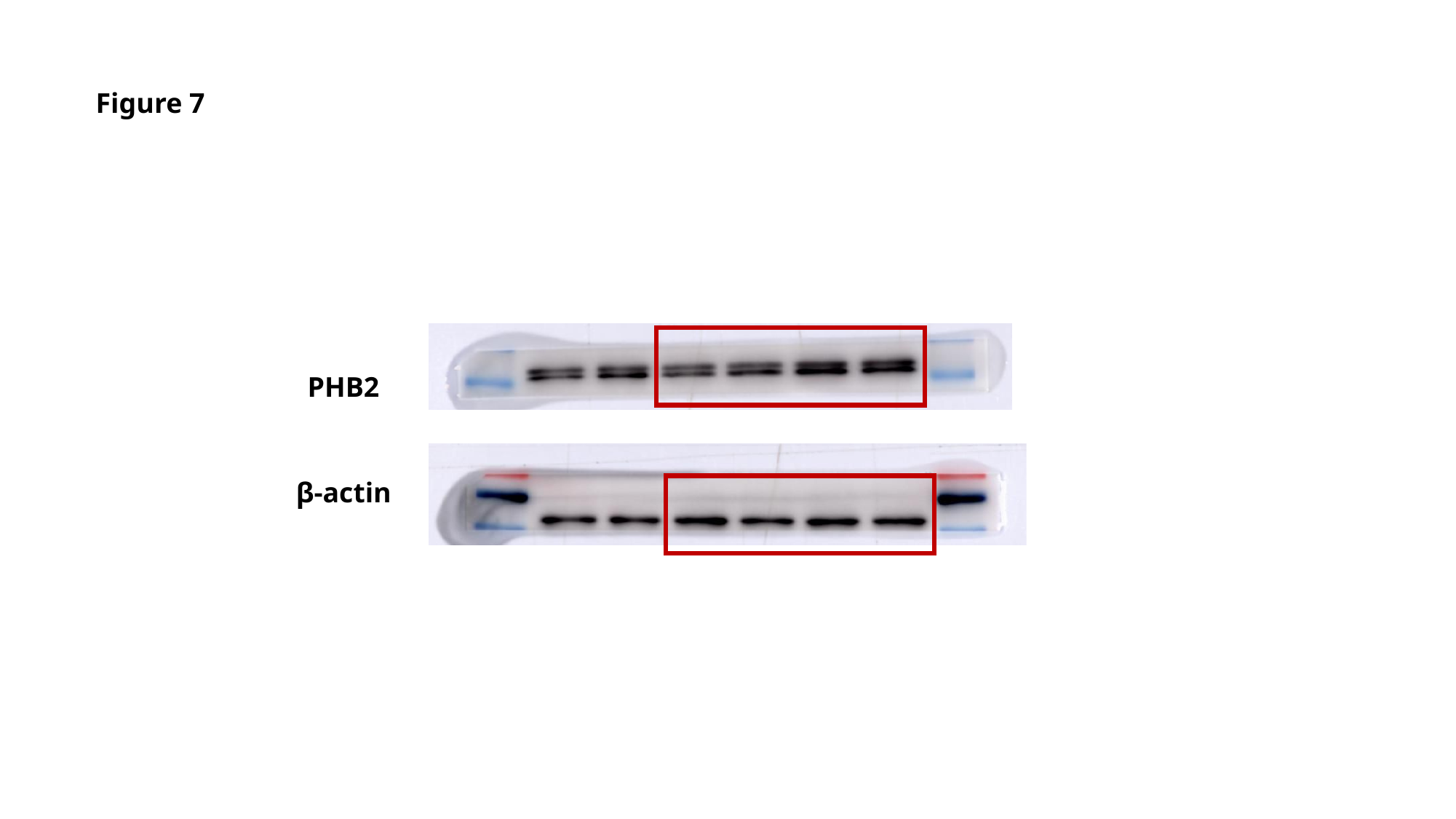

Figure 7
PHB2
β-actin

## Slide 9
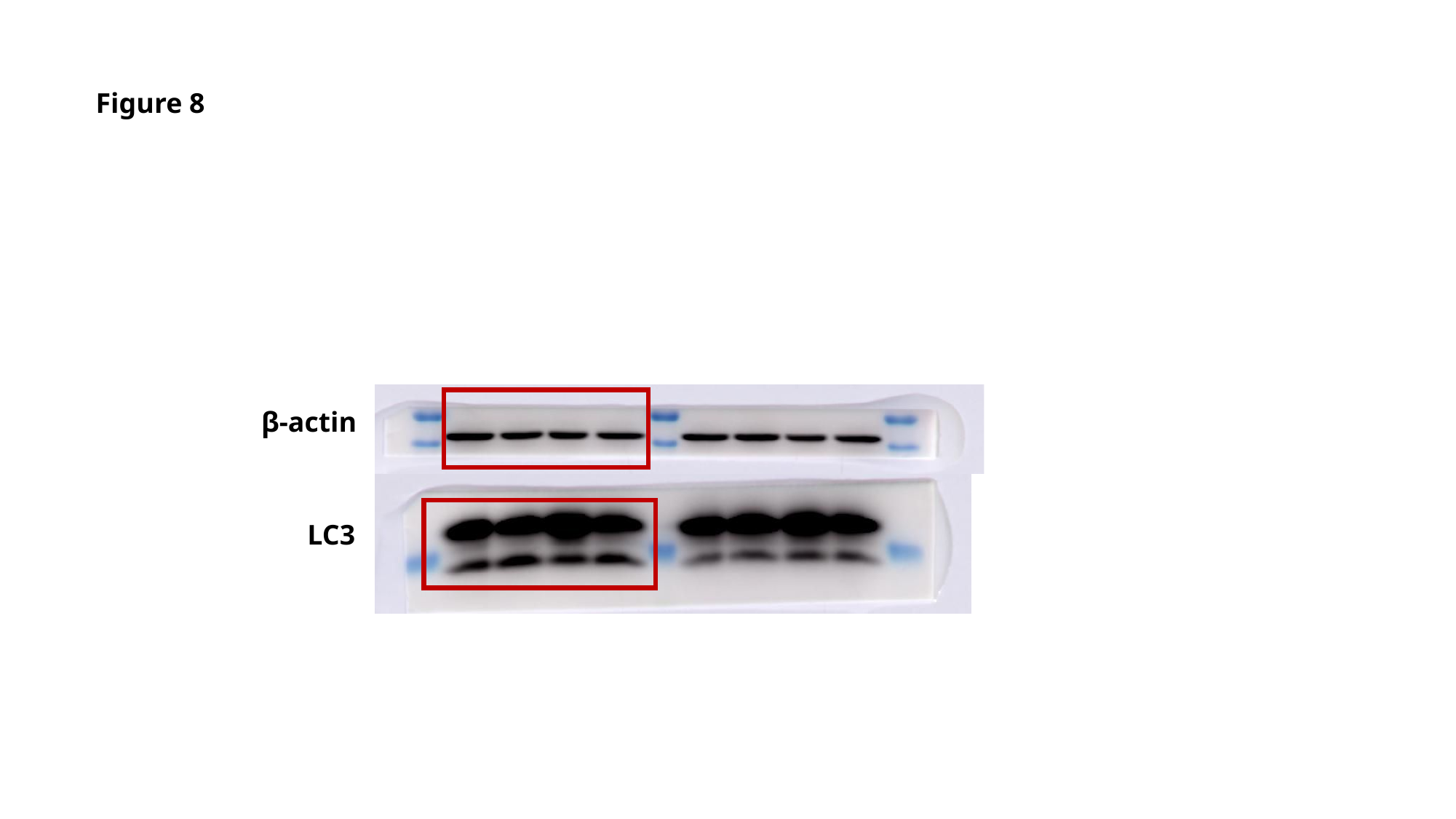

Figure 8
β-actin
LC3
